# Determination of Soil Pore Water Salinity Using an FDR Sensor Working at Various Frequencies up to 500 MHz

**DOI:** 10.3390/s120810890

**Published:** 2012-06-18

**Authors:** Andrzej Wilczek, Agnieszka Szypłowska, Wojciech Skierucha, Jolanta Cie(x0015B)la, Viliam Pichler, Grzegorz Janik

**Affiliations:** 1 Institute of Agrophysics, Polish Academy of Sciences, ul. Do(x0015B)wiadczalna 4, 20-290 Lublin, Poland; E-Mails: a.wilczek@ipan.lublin.pl (A.W.); w.skierucha@ipan.lublin.pl (W.S.); j.ciesla@ipan.lublin.pl (J.C.); 2 Department of Natural Environment, Faculty of Forestry, Technical University Zvolen, T. G. Masaryka 24, 960 53 Zvolen, Slovakia; E-Mail: pichler@vsld.tuzvo.sk; 3 Institute of Environmental Protection and Development, Wroclaw University of Environmental and Life Sciences, Pl. Grunwaldzki 24, 50-363 Wrocław, Poland; E-Mail: grzegorz.janik@up.wroc.pl

**Keywords:** dielectric spectroscopy, frequency-domain reflectometry, complex dielectric permittivity, salinity index approach, soil salinity, soil water content

## Abstract

This paper presents the application of a frequency-domain reflectometry (FDR) sensor designed for soil salinity assessment of sandy mineral soils in a wide range of soil moisture and bulk electrical conductivity, through the determination of soil complex dielectric permittivity spectra in the frequency range 10–500 MHz. The real part of dielectric permittivity was assessed from the 380–440 MHz, while the bulk electrical conductivity was calculated from the 165–325 MHz range. The FDR technique allows determination of bulk electrical conductivity from the imaginary part of the complex dielectric permittivity, without disregarding the dielectric losses. The soil salinity status was determined using the salinity index, defined as a partial derivative of the soil bulk electrical conductivity with respect to the real part of the soil complex dielectric permittivity. The salinity index method enables determining the soil water electrical conductivity value. For the five sandy mineral soils that have been tested, the relationship between bulk electrical conductivity and the real part of dielectric permittivity is essentially linear. As a result, the salinity index method applied for FDR measurements may be adapted to field use after examination of loam and clayey soils.

## 1. Introduction

Accurate and reliable estimation of soil salinity, defined as the electrical conductivity of soil water extract or saturated soil water extract, is a very important issue, especially in arid regions, where salinity of the soil may increase significantly and pose a danger to plants [1,2]. Soil salinity measurement is rather complicated as it is often affected by factors producing measurement errors, e.g., temperature, moisture and texture of the soil. Therefore, the development of new measurement methods and equipment for evaluating soil salinity status is still an active field of research. The ideal measurement tool for soil salinity status should read the majority of influencing components at the same time and in the same location as quickly as possible to register the momentary values of interest. This is done by incorporating various sensors in a single unit for insertion into the measured material [3,4] or by selective analysis of sensors' outputs for discrimination of various quantities like soil water content and electrical conductivity by time domain reflectometry [5]. The discrimination techniques sometimes require increased accuracy of measurement and additional calibrations, like calibration of TDR probes for low soil water content measurement [6]. Application of indirect measurement sensors based on soil dielectric properties, *i.e.*, TDR and FDR sensors, has opened new possibilities through the ease of integrating the sensing elements of soil water content, electrical conductivity and temperature in one small measurement probe. The concept of the soil salinity index *X_s_*, describing soil salinity status, was introduced by Malicki and Walczak [7] as one of many applications of the TDR technique for nondestructive and simultaneous measurement of water state and its transport in soil [8]. The salinity index was defined as a partial derivative of the soil bulk electrical conductivity *C_b_* with respect to the soil bulk or apparent dielectric permittivity *ε_b_* (Equation (1)), where both variables were determined from the TDR waveform at the same time and on the same soil volume:

(1) $$X_{s}=\frac{\partial C_{b}}{\partial {ɛ}_{b}}$$

Soil bulk dielectric permittivity and electrical conductivity were determined from the velocity of the TDR pulse and its attenuation, respectively, when traveling along the TDR parallel waveguide inserted in the soil [5,9]. Malicki and Walczak [7] showed that the *X_s_* value depends primarily on soil salinity and soil texture. Soil salinity can thus be uniquely determined from the salinity index *X_s_* and the sand content of the soil. Their experimental evidence showed that the soil salinity index as a function of soil electrical conductivity of the electrolyte is independent of the soil volumetric water content for values of water content above 0.2.

The application of the TDR technique in calculating the salinity index includes simplifications that can affect the final result. The value of *ε_b_* approximates the real part *ε*′ of the complex dielectric permittivity and is dependent on the frequency *f* of the applied external electric field. Also, the electrical conductivity, *C_b_*, calculated from the attenuation of the TDR pulse, does not take into account the dielectric loss *ε_d_*. The complex dielectric permittivity ε*, presented by Equation (2), comprises the real part that describes the energy storage in dielectric medium, and the imaginary part that measures the energy losses. These losses may be caused by dissipation of energy during polarization processes (dielectric loss) and by electrical conductivity of the material:

(2) ɛ∗(f)=ɛ′(f)−j(ɛd+Cb2πfɛ0)=Re(ɛ∗)−jIm(ɛ∗)

where *ε_0_* = 8.85 × 10^−12^ F m^−1^ stands for dielectric permittivity of free space.

The dependence of the complex dielectric permittivity of soils on the frequency of the applied electrical field has been discussed in literature; however, the applied frequency was not defined [10], the interpretation was limited to 50 or 150 MHz [11,12] or the frequency spectrum was obtained from the TDR waveform [12].

It was shown [10] that the separation of real and imaginary parts of the complex dielectric permittivity would remove the influence of the conductivity effect on the water content calibration of the FDR technique compared with TDR. The application of the broadband FDR technique allows measuring the real and imaginary parts of the complex dielectric permittivity of soil separately and independently. Consequently, for water content measurements it is possible replace the bulk dielectric permittivity (the product of TDR) with the real part of the dielectric permittivity determined by FDR in the frequency range near 400–500 MHz, where the influence of electrical conductivity on the real part of dielectric permittivity is negligible. Another reason for developing FDR devices for measuring soil moisture and salinity is that the TDR meters require the application of steep pulses with very short rising times (at least several times less than a nanosecond), which significantly contributes to the cost of these devices.

The aim of the paper is to determine the bulk electrical conductivity and soil pore water electrical conductivity of selected soil samples based on independent measurements of the real and imaginary parts of the complex dielectric permittivity using the FDR technique in the frequency range of 10 MHz to 500 MHz. The received FDR-based linear salinity index is compared with the Malicki and Walczak [7] TDR-based salinity index presented in Equation (1). The quadratic FDR-based salinity index model, which takes into account possible quadratic contributions to the *C_b_ vs. ε′* relation, is presented and its performance is compared to the linear model. The possibility of obtaining the soil pore water electrical conductivity value from a single FDR measurement of *C_b_* and *ε′*, necessary for adapting the FDR-based salinity index approach to field use, is also evaluated.

## 2. Materials and Methods

zThe tested material included five mineral soils that were air dried and put through a 2 mm sieve. The basic physical characteristics of these materials are given in Table 1. The soils were collected from the Ap layer, 20–30 cm below the surface. The material dry bulk density was determined in the laboratory in 10-fold repetitions by weighing soil samples of known volumes, drying them at 105 °C for 24 hours and again weighing [13]. The respective values from Table 1 are calculated for a confidence level of 95%. Having determined the material bulk density, it was possible to calculate its mass in a measurement container of 120 cm^3^ volume (cylinder; diameter: 4.6 cm, height: 7 cm), which helped to prepare appropriate soil samples with variable water content ranging from approximately 10% to near saturation. The soil material of various moisture levels was packed into the containers in small portions and pressed with a 0.2 kg rubber hammer to achieve homogenous density distribution in the soil sample [7]. There was no intention of achieving predefined values of soil sample density, as the experiment tested only the idea of the FDR-determined salinity index. Five soil materials were tested. Each soil was wetted with four solutions (distilled water and three KCl solutions, presented in Table 2), to seven soil moisture content levels. There were 35 pots with soil samples for each wetting solution, giving a total number of 140 pots. FDR measurements were performed three times on each sample.

The values of saturation water content by mass for the selected soils were determined using capillary rise. Three samples of each soil of a known dry weight placed in measurement containers with holes at the bottom, were gathered in separate plastic bowls with porous bottom sides. The containers with soil, covered to minimize excessive evaporation, were immersed for 72 hours in distilled water allowing capillary rise to achieve saturation. Then they were weighed to determine the mass of water saturating each soil sample. Finally, after simple calculations it was possible to determine the mass of water for adding to the 120 cm^3^ volume of air-dry soil to achieve soil samples of a desired water content, from 10% to near saturation.

On the basis of generally available conversion tables and pilot measurements of soil electrical conductivity performed by a TDR meter, three KCl solutions and distilled water were prepared for wetting soil samples (Table 2). Application of KCl solutions of various electrical conductivity *C_s_* enabled changing the real and imaginary parts of dielectric permittivity of the tested material to achieve the assumed range of variability of the soil electrical conductivity of 100–400 mS m^−1^, and of soil water content—from air dryness to near saturation. The samples of chosen soils fully filled 120 cm^3^ containers (plastic cylinder 4.6 cm in diameter and 7 cm in height) equipped with a sealed cover, and wetted by distilled water and the KCl solutions.

The material samples in containers were mixed with distilled water and KCl solutions, covered with a sealing cap, weighed and conditioned at 40 °C for 72 hours to ensure uniform water content in the sample volume. Then, after leaving the soil containers for several hours at room temperature, the filled containers were again weighed to make weight corrections caused by possible water evaporation loss. Next, the containers were opened to perform FDR measurements of the complex dielectric permittivity of the material. Each FDR measurement was made three times by inserting the probe rods in various locations of the material in the container. All measurements were made in a laboratory with a controlled temperature 21 ± 1°C.

### 2.1. FDR Probe

The applied FDR sensor, measurement details and calibration techniques were described earlier by Skierucha and Wilczek [17]. The probe consisted of a parallel waveguide with two steel rods, similar to probes used with the TDR instruments developed at the Institute of Agrophysics PAS in Lublin, Poland (easytest.ipan.lublin.pl), but shorter, *i.e.*, 3 cm against 10 cm. The probe with such short rods applied with a TDR meter would require even shorter rising time of the pulse, which is possible in extremely sophisticated and expensive TDR devices. With a probe of this type, the measured quantities pertained to a greater volume of the sample under test than in the case of an open-ended coax probe [18]. The soil is a highly inhomogeneous, multiphase material and testing of it provides many challenges, as the measurement result taken at a given point in the sample could differ from the result obtained at another point. Therefore, the open-ended coax probe is impractical in this case, as it would introduce significant errors due to the inhomogeneity of the material. A vector network analyzer (VNA), type ZVCE from Rohde and Schwarz, was used for measuring the complex reflection coefficient *S*_11_ of the signal reflected from the probe inserted into the sample. This reflection coefficient is defined as:

(3) $$S_{11}=\frac{Z_{p}({ɛ}^{*})-Z_{c}}{Z_{p}({ɛ}^{*})+Z_{c}}$$

where *Z_p_* is the impedance of the probe and *Zc* = 50 Ω. The impedance of the probe depends on the complex dielectric permittivity of the material in which the sensor was inserted. Therefore, the measured reflection coefficient is also a function of the dielectric permittivity of the sample. It is possible to obtain the inverse relation: ε* = *f*(*S*_11_), which allows determining the complex dielectric permittivity from the measured reflection coefficient for each applied frequency. For calibration purposes, measurements of short circuit, open (air) and acetone were used, which reduced the measurement errors [19].

## 3. Results and Discussion

### 3.1. Complex Dielectric Permittivity

**Figure 1.**
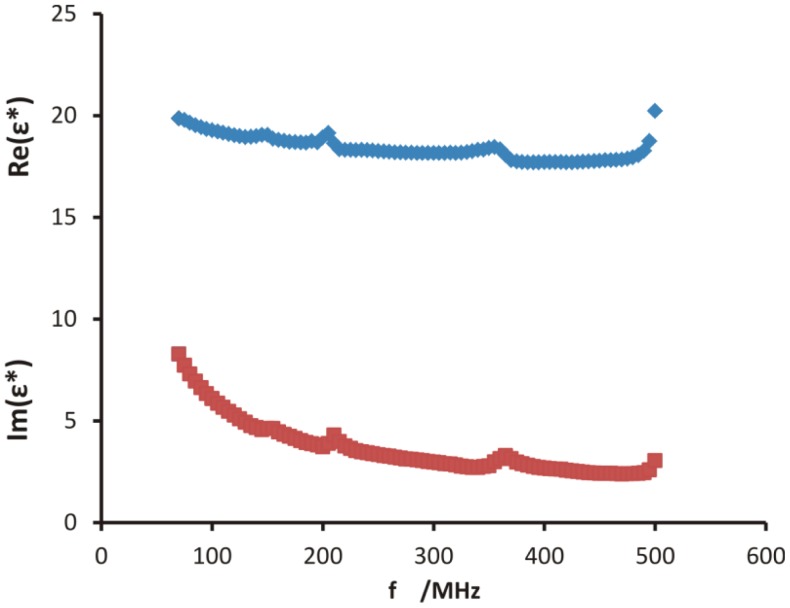
Real and imaginary parts of the complex dielectric permittivity of a sample of soil no. 601 wetted with distilled water to approximately 50% of saturation water content.

The FDR measurement technique allowed determining the real and imaginary parts of the dielectric permittivity directly and independently. Figure 1 presents the real and imaginary parts of the dielectric permittivity measured for a sample of soil no. 601 wetted with distilled water to approximately 50% of the saturation water content. The other soil samples were tested in a similar way. On the graph one can notice artifacts located near frequencies of 150, 205, 360 and 500 MHz for both parts of ε*. They are related to resonances in the experimental set-up, due mostly to the length of the coax cable connecting the sensor with the VNA; the details and discussion are presented in [17]. This was later confirmed by introducing a magnetic shield on the coax cable, which substantially decreased the effect of the artifacts. The frequencies for which the artifacts occur will be excluded from further analysis, without negatively influencing the obtained results.

The real part of the dielectric permittivity is strongly related to the soil water content, namely the square root of the real part of the dielectric permittivity (the refractive index) depends linearly [20] on the volumetric water content, measured by the standard bulk density plus thermogravimetric soil water content method. This dependence for results obtained by the FDR probe used in this experiment is confirmed in [18]. To minimize the effect of electrical conductivity of the sample on the real part of dielectric permittivity, the values of the real part of the dielectric permittivity were taken for the frequency range 380–440 MHz [17].

### 3.2. Electrical Conductivity of Soil Samples

The imaginary part of the complex dielectric permittivity may be used to infer the bulk electrical conductivity of the sample. According to Equation (2), the imaginary part of the complex dielectric permittivity ε* is related to bulk electrical conductivity of the sample *C_b_*

(4) Im(ɛ∗)=ɛd+Cb2πɛ0f

One can multiply both sides of the above equation by the frequency, so that the whole relation is a linear function of *f*:

(5) Im(ɛ∗)⋅f=ɛdf+Cb2πɛ0

**Figure 2.**
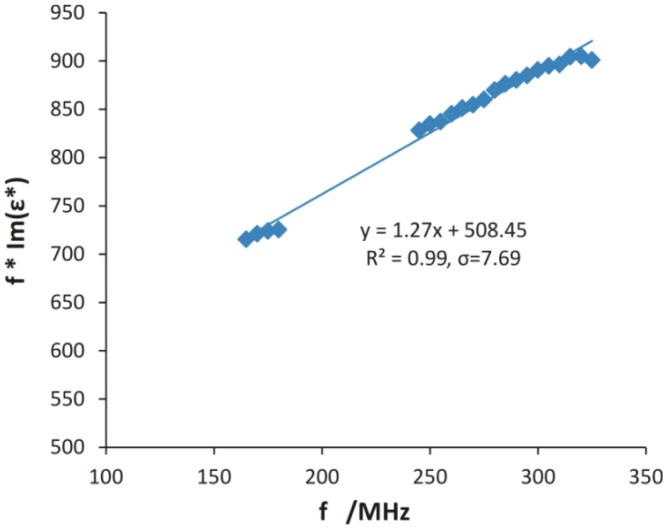
Determination of bulk electrical conductivity of a sample of soil no. 601 wetted with distilled water to 50% of saturation water content; *C_b_* is directly proportional to the intercept, according to Equation (5). The regression equation, *R*^2^ and standard error of regression are given on the plot. The standard error of determination of *C_b_* is about 2%.

Assuming that *ε_d_* is not frequency dependent in the analyzed frequency range up to 500 MHz, one can find the values of *C_b_* by fitting a straight line into a plot of the above function, as shown in Figure 2. The regression equation, coefficient of determination *R*^2^ and standard error of regression *σ*, defined as the square root of the sum of the squared residuals divided by the number of degrees of freedom, were presented on the graph. To calculate bulk electrical conductivity with the artifacts removed, frequencies from 165 to 180 and from 245 to 325 MHz were selected. In this frequency range, which is narrow enough and distant from relaxation frequencies of various polarization mechanisms which may occur in the tested soils, the assumption that the dielectric loss *ε_d_* does not depend on frequency is reasonable and gives good fits, as presented by the example in Figure 2.

This procedure was applied to determine the bulk electrical conductivity *C_b_* of all tested soil samples.

### 3.3. Salinity Index and Electrical Conductivity of Soil Water

**Figure 3.**
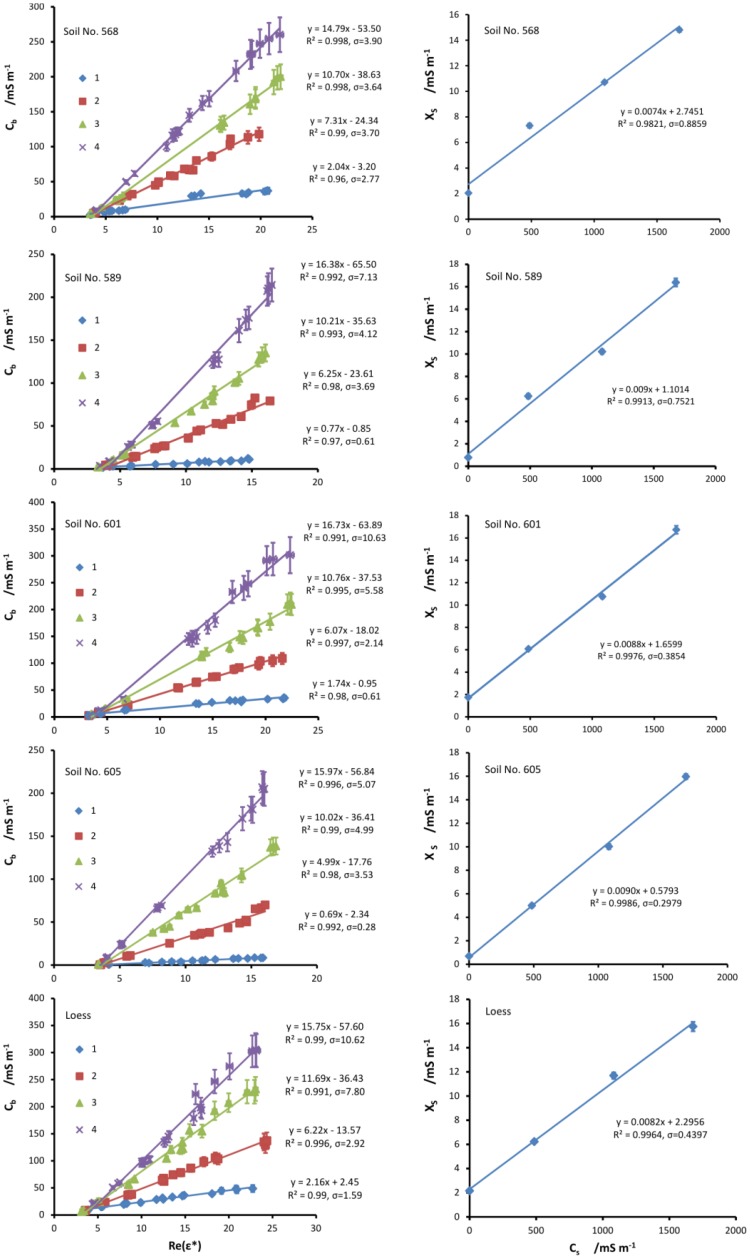
On the left: bulk electrical conductivities of all measured soil samples *vs.* the real part of the dielectric permittivity. On the right: salinity index *X_S_* calculated as a slope of a linear *C_b_ vs. ε′* relation, with respect to the conductivity of the moistening solutions *C_s_*. Regression equations, *R*^2^ and standard errors of regressions are given on the plots.

In order to test the concept of salinity index introduced by Malicki and Walczak [7] with respect to the FDR measurements of the real and imaginary parts of the dielectric permittivity of soils, the electrical conductivity *C_b_* of each sample versus the real part of dielectric permittivity *ε′* = Re(ε*) was plotted for all moistening solutions and a straight line was fitted through each set of data points. The results are presented on the left panel of Figure 3. The regression equation, *R*^2^ and standard error of regression *σ*, defined as in the previous section, were presented on the graphs. For all points, error bars are present. The vertical error bars represent the standard error of determination of *C_b_*. Taking into account these errors and high values of *R*^2^ (as presented on the graph), it transpires that for all tested samples the relations between *C_b_* and *ε′* are essentially linear. Thus, the salinity index defined as:

(6) $$X_{S}=\frac{\partial C_{b}}{\partial {ɛ}^{\prime }}$$

is actually the slope of the fitted line in the *C_b_ vs. ε′* graph and, for linear *C_b_ vs. ε′* dependence, is independent of the water content. The difference between Equation (6) and Equation (1), which presents the salinity index defined for TDR measurements, is the use of the real part of the dielectric permittivity instead of the apparent dielectric permittivity. In addition, the electrical conductivity in Equation (6) was determined from the frequency spectrum of the imaginary part of the dielectric permittivity as described in Section 3.2, while in Equation (1) the quantity *C_b_* actually denoted bulk (or apparent) electric conductivity determined from attenuation of the TDR pulse with the assumption that the dielectric loss *ε_d_* = 0.

The relations between the salinity index and moistening solution conductivity *C_s_* for the examined soils are presented on the right panel of Figure 3. Each value of *X_s_* was determined from the slope of the regression equations of *C_b_* against *ε′*, which were shown on the left panel of Figure 3. It was found that *X_s_* depends linearly on the electrical conductivity of the moistening solution *C_s_*. When the salinity index *X_s_* and the slope *l* of the *X_s_ vs. C_s_* relation are known, one can calculate the conductivity of soil water from the following formula:

(7) $$C_{w}=\frac{X_{s}}{l}$$

One may notice that for the samples wetted with distilled water the salinity index is equal to some initial value *X_SI_* ≠ 0, due to some residue conductivity of ions dissolved from dry soil. As was shown in [7], this residue conductivity *C_r_* may be found by extrapolating the *X_s_ vs. C_s_* relation to the horizontal axis. Then:

(8) $$C_{r}=\frac{X_{SI}}{l}$$

Therefore, one may expect that the electrical conductivity of soil water *C_w_* is a sum of the residue electrical conductivity and the conductivity of the moistening solution:

(9) $$C_{w}=C_{r}+C_{S}$$

This assumption will be tested in a subsequent part of this paper.

### 3.4. Salinity Index Method for Field Use

To calculate the salinity index using the method presented above, it is necessary to take a series of measurements of the same soil moistened with the same solution to various water contents. Obviously, this procedure has little practical use, since a measure of soil salinity applicable for field conditions should provide an accurate estimate based on a single measurement of a single soil sample. However, similarly to the method shown in [7], if the relation *C_b_ vs. ε′* is linear, one need not take a series of measurements of samples of different water contents of the soil under question to determine the value of the salinity index—the measurement of complex dielectric permittivity of a single sample will suffice. It transpires that the lines from the left panel of Figure 3, fitted into the (*ε′,C_b_*) data points, for a given soil and all applied solutions, cross at certain limiting values of the real part of the dielectric permittivity and bulk electrical conductivity. These limiting values are denoted *ε_I_* and *C_I_*, respectively. Once these values are known for a given soil, it is possible to calculate the salinity index by determining the value of the partial derivative from an appropriate difference quotient:

(10) $$X_{s}=\frac{\partial C_{b}}{\partial ɛ\prime }≅\frac{\Delta C_{b}}{\Delta {ɛ}^{\prime }}=\frac{C_{b}-C_{I}}{{ɛ}^{\prime }-{ɛ}_{I}}$$

with the use of the values of *ε′* and *C_b_* measured from a single sample of previously unknown salinity and water content. Therefore, combining Equations (7) and (10), the electrical conductivity of soil pore water may be calculated from the following relation:

(11) $$C_{w}=\frac{C_{b}-C_{I}}{l({ɛ}^{\prime }-{ɛ}_{I})}$$

To apply the formula above in the field, it is necessary to know the values of *ε_I_* and *C_I_* for a given soil. The values, obtained in this experiment for the five tested soils, depend on the soil properties. It was found that *ε_I_* depends on the specific surface *s* of the soil under question, with regression equation given by *ε_I_ =* 0.02*s* + 3.44 with *R*^2^ = 0.99, where *s* is given in units from Table 1. On the other hand, for the tested soils *C_I_* is affected by the clay content *c*. The appropriate regression equation is *C_I_* = 0.65*c* + 3.23, with *R*^2^ = 0.89 and clay content given in units from Table 1. However, before these equations can be used in the field for an unknown soil, it is necessary to perform laboratory measurements of a greater number of soils with different texture and other properties to obtain more reliable regression equations. Because of the limited number of the tested soils, the regression equations on *ε_I_* and *C_I_* are included only to present the variability of these parameters with the soil texture and are not used in further calculations. The accuracy of the values of *ε_I_* and *C_I_* is especially important for soils which are either very dry, or have low bulk electrical conductivity. This is because from the form of the last term of Equation (10) it transpires that for samples with values of *C_b_* and *ε′* close to *C_I_* and *ε_I_*, respectively, the error of the final value is the most affected by the errors of all these four quantities.

To calculate the electrical conductivity of soil water *C_w_* from the salinity index *X_S_* in the field conditions from Equation (11), the value of the parameter *l* needs to be known beforehand too. As was the case with *ε_I_* and *C_I_*, the parameter *l* also depends on the soil properties. For the five tested soils, the best fit was obtained for a straight line of equation *l* = −0.00008*c* + 0.009, where *c* is the clay content, as for *C_I_*. However, *R*^2^ equals only 0.56. It is obvious that in order to minimize the error of *C_w_* determined in field conditions, additional soils of different properties (especially loam and clayey soils) should be tested in order to achieve better regression equations.

### 3.5. Comparison of Linear Models of Salinity Index

**Figure 4.**
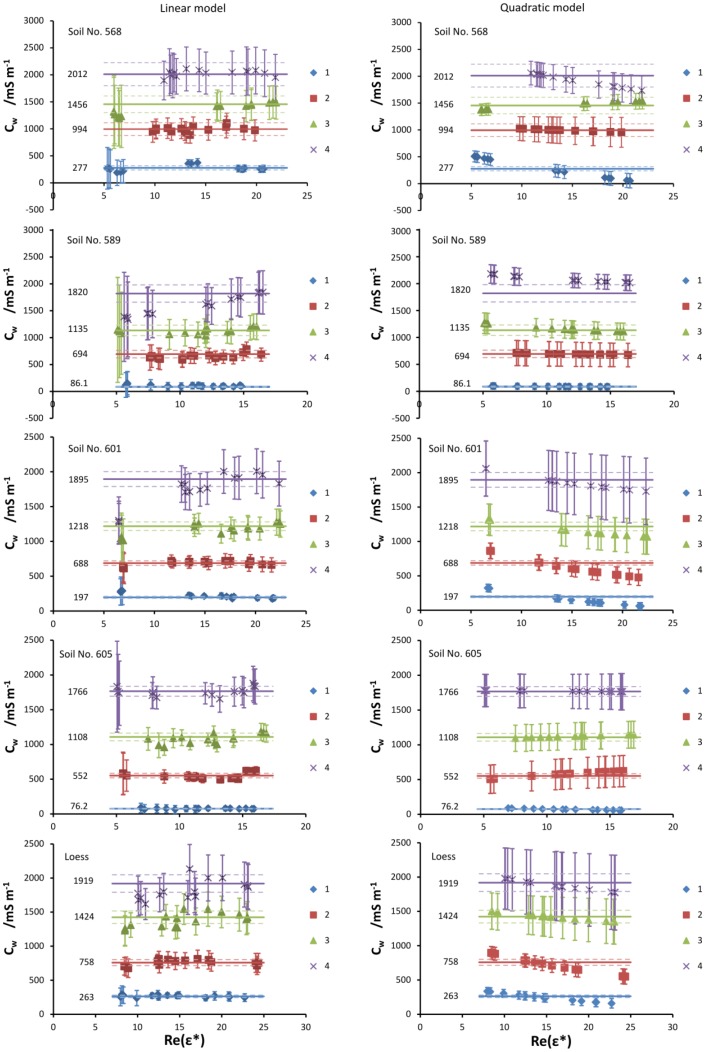
Soil water conductivities *C_w_* versus *ε′ =* Re(ε*) for linear and quadratic salinity index models for all moistening solutions. Solid lines represent *C_w_* values calculated with the use of *X_S_* obtained from the slopes of *C_b_ vs. ε′* linear relations.

On the left panel of Figure 4, the values of soil water electrical conductivity *C_w_* for all tested soils are shown. The “linear model” phrase at the top of the figure means that the values of *C_w_* were calculated with the assumption that the relation *C_b_ vs. ε′* is linear as in the left panel of Figure 3. Possible quadratic effects are taken into account in the “quadratic model” elaborated in the next section. The straight lines on all graphs represent the values of *C_w_* (these values are also written on the right side of the lines) calculated from Equation (7) with the use of the salinity index *X_S_* obtained from the regression equations from the left panel of Figure 3 and *l* values determined from the slopes of the regression lines from the right panel of Figure 3. Since the salinity index calculated from the slope of a straight line fitted through the data points obtained from the whole series of samples moistened to various water contents (to various values of *ε′*) by the same solution, the value of *C_w_* is from definition independent of the water content (or *ε′*, equivalently). The errors of *C_w_*, represented by the dashed lines, depend on the standard error of determination of *X_S_* as a slope of the fitted straight line and on the standard error of determination of slope *l* of the *X_S_ vs. C_S_* relation.

The data points shown on the left panel of Figure 4 represent the values of *C_w_* calculated from Equation (11) with the use of the salinity index obtained from Equation (10), simulating field conditions (which may be named the “linear field model”). These values are obtained for each sample independently. The values of *l* were taken as in the previous linear model. The errors of *C_w_*, represented by the error bars, depend on the standard errors of all quantities used in Equation (10), as well as on the standard error of the parameter *l*. As can be seen, these errors are much greater than in the previous case, where *X_S_* from regression were used. Because of the form of Equation (10), as already mentioned, the errors may approach unreasonably high values for dry soils, which may be seen on the plots. Because of this, it was not possible to obtain reliable values of *C_w_* for the two samples with the least water content for each soil and moistening solutions; therefore, these values have not been presented in Figure 4.

The values of *C_w_* obtained from the linear field model are roughly independent of the water content and agree with the values calculated from *X_S_* obtained from regression. A notable exception to this is soil no. 589 moistened with solution no. 4 (the most concentrated KCl solution), as seen on the left panel of Figure 4. However, the values of *C_w_* calculated from *X_S_* obtained from regression are still encompassed within the error bars of the field model values.

### 3.6. Quadratic Model of Salinity Index

On the left panel of Figure 3, straight lines were fitted to approximate *C_b_ vs. ε′* relation for all measurement series. However, some possible quadratic effects may be lost. In order to assess the possible impact of quadratic terms on the *C_w_* values, a quadratic model of the salinity index has been examined.

Even when the dependence *C_b_ vs. ε′* is not linear, it is still possible to use the salinity index defined as in Equation (6). With the quadratic relation:

(12) $$C_{b}=a{\left({ɛ}^{\prime }\right)}^{2}+b{ɛ}^{\prime }+c$$

which was fitted into the (*ε′, C_b_*) data points for all measurement series (not presented on figures), the salinity index calculated as a derivative takes the form:

(13) $$X_{S}=2s{ɛ}^{\prime }+b$$

and is obviously dependent on the real part of dielectric permittivity, and thus on the water content. To calculate electrical conductivities of soil water with the use of Equation (7), it is necessary to obtain slopes *l* of the *X_S_ vs. C_S_* relations, for each value of *ε′*. Fortunately, it transpired that the *l vs. ε′* is linear (*R*^2^ = 0.9988 for the worst fit for soil no. 568), which enables calculating *C_w_* for arbitrary values of *ε′*. The values of electrical conductivity of soil water obtained with the use of the quadratic model of the salinity index are presented on the right panel of Figure 4. For comparison purposes, straight lines representing *C_w_* values calculated from *X_S_* obtained from regression from the linear model are also presented on the plots. One may notice that the values of *C_w_* either remain constant with *ε′* (soil no. 605, 589, two series of soil no. 568) or decrease with the increase in *ε′*. Also, for most measurement series, the values of *C_w_* obtained by all three approaches are comparable.

The errors of *C_w_* calculated from the quadratic model, which depend mostly on the errors of the salinity index calculated from Equation (13), are quite high—with few exceptions, comparable to or even higher than for the linear field model, as can be seen in Figure 4 and Table 3. Table 3 shows a comparison of relative errors of soil water conductivity *C_w_*, determined from the salinity index obtained from: (i) regression from the linear model, (ii) calculated from Equation (10) (linear field model) and (iii) calculated from the quadratic model (Equation (13)). The presented errors pertain to *C_w_* obtained for loess for all four applied moistening solutions, for samples with similar values of the real part of dielectric permittivity. For other tested soils, these errors behave similarly. This suggests that the inclusion of the quadratic term in the *C_b_ vs. ε′* relation does not improve the *C_w_* estimation.

Generally, only the linear field model may exhibit greater errors for *C_w_* than the quadratic one; however, not for all cases, as can be seen in Figure 4. Furthermore, because the salinity index in the quadratic model depends on water content, it would be very difficult to adapt it for field use. Because of this, the quadratic model does not present any significant improvement over the linear models for the tested soils.

### 3.7. Conductivity of the Moistening Solution

To further test the linear salinity index model, introduced by Malicki and Walczak in [7], one may calculate the conductivity of the moistening solution *C_S_* from Equation (9), by subtracting the residual conductivity *C_r_* (taken as conductivity of soil water for samples wetted with distilled water) from the conductivity of soil water *C_w_*:

(14) $$\begin{array}{cc} C_{S}=C_{w}-C_{r}, & C_{r}\equiv C_{w(\text{distilled water})} \end{array}$$

**Figure 5.**
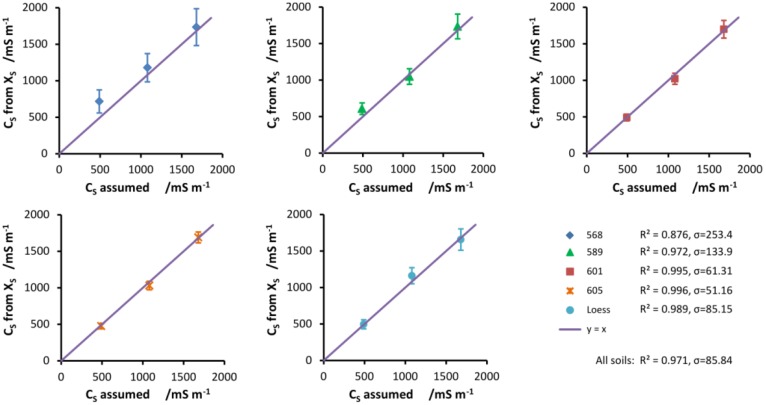
Conductivities of the moistening solutions calculated from the linear salinity index model *vs.* assumed conductivities for all tested soils. The straight line has a slope equal to 1 and corresponds to the perfect agreement.

The results can be then compared with *C_S_* assumed from the molarity of the solutions (Table 2). The values corresponding to distilled water are equal to zero from definition. The results, calculated for the salinity index obtained as a slope of *C_b_ vs. ε′* relation (linear model), are presented in Figure 5. The values of *R*^2^ and the standard errors *σ* presented in the figure describe how well the assumed values of *C_S_* explain the calculated quantities. As can be seen from the values, the agreement is very good. The worst case is soil no. 568, which exhibits both high errors in the calculated *C_S_*, as shown by the error bars, and the worst fit for the assumed values. The best case is soil no. 605, which has 95% sand fraction. This occurrence, along with the errors of *C_w_* presented in Figure 4, shows that the salinity index method is generally the most reliable for soils with the highest sand content. Because the calculated electrical conductivities of the moistening solutions have been determined from linear model, the values corresponding to distilled water are equal to zero from definition

## 4. Conclusions

The present study deals with the measurement of soil salinity using the FDR technique. It enables directly obtaining real and imaginary parts of complex dielectric permittivity. The obtained Equation (11) enables assessment of the soil pore water salinity from FDR measurements of soil bulk electrical conductivity and soil dielectric permittivity. Some of the parameters of this equation depend on soil properties, mostly clay content and specific surface area. Because of the coarse texture of the soil samples, those parameters have been determined just for sandy soils, and much research is needed for loam to clayey soils. In order to remove the influence of other physical factors and processes on the measured quantities, determination of water content was performed from the real part of dielectric permittivity in the frequency range 380–440 MHz, while the soil salinity was determined from the imaginary part of the dielectric permittivity for lower frequencies.

The salinity index method, introduced in [7] for soil salinity estimation with the use of the TDR measurements, may still be applicable when the measurements are performed using the FDR technique. However, adaptation of the salinity index method for field use would require a good deal of work, because of the necessity to test wider varieties of soils with the application of more moistening solutions, in order to increase the accuracy of determining the electrical conductivity of soil water.

The linear salinity model by definition gives electrical conductivity of soil pore water independent of the real part of dielectric permittivity and thus of water content. This ignores the possible ion adsorption and desorption processes occurring on the surfaces of the soil solid particles. Even though the chosen soils were sandy, they differed in specific surface area values (as shown in Table 1). Therefore, the application of the quadratic salinity index model, in which the salinity index depends on water content, could possibly detect adsorption effects. This was not so for the tested soils. However, the methodology presented in this paper will be tested in the future with soil samples with larger specific surface area values (loam and clayey soils).

## Figures and Tables

**Table 1.** Selected physical characteristics of tested material; RSD stands for relative standard deviation (standard deviation/mean value).

**Name**	**Bulk density (kg·m^−3^)**	**Specific surface** ^a^ **(m^2^·g^−1^)**	**Saturation water content by mass (%)**		**Texture (FAO)** ^b^ **(%)**		

			**Average**	**RSD**	**sand**	**silt**	**clay**
568	1,415 ± 3	34	37.2	0.03	58	31	11
589	1,697 ± 4	10	22.4	0.04	88	11	1
601	1,380 ± 4	31	38.7	0.02	60	34	6
605	1,635 ± 7	10	23.8	0.07	95	4	1
loess	1,382 ± 4	30	37.6	0.01	55	29	16

a Water vapor adsorption method [14].

b Data taken from Glinski *et al.* [15].

**Table 2.** Parameters of KCl solutions applied for wetting the measured material (values at temperature 21 °C). Assumed *C_s_* values were calculated from molarity [16] and confirmed by measurements.

**Label of solution**	**KCl molarity (mol·dm^−3^)**	**Assumed *C*_S_ (mS·m^−1^)**
1	0.000	–
2	0.042	490
3	0.093	1,080
4	0.150	1,680

**Table 3.** Conductivities of water content *C_w_* calculated from linear, linear field and quadratic salinity index models, with respective errors, for four moistening solutions, for samples of loess with similar values of the real part of the dielectric permittivity.

No. of solution	Re(ε*)	*C*_w_ (linear model) (mS m^−1^)	**Relative error (%)**	*C*_w_ (linear field model) (mS m^−1^)	**Relative error (%)**	*C*_w_ (quadratic model) (mS m^−1^)	**Relative error (%)**
1	13.6	263	**6.9**	278	**13.5**	255	**23.5**
2	13.6	758	**5.8**	801	**14.7**	756	**12.8**
3	13.4	1,424	**6.5**	1,434	**12.4**	1,441	**19.7**
4	13.1	1,919	**6.7**	1,796	**15.1**	1,921	**24.7**
